# Functional genetic variant in the Kozak sequence of WW domain-containing oxidoreductase (WWOX) gene is associated with oral cancer risk

**DOI:** 10.18632/oncotarget.12082

**Published:** 2016-09-16

**Authors:** Hsin-Lin Cheng, Yu-Fan Liu, Chun-Wen Su, Shih-Chi Su, Mu-Kuan Chen, Shun-Fa Yang, Chiao-Wen Lin

**Affiliations:** ^1^ Institute of Medicine, Chung Shan Medical University, Taichung, Taiwan; ^2^ Department of Biomedical Sciences, Chung Shan Medical University, Taichung, Taiwan; ^3^ Whole-Genome Research Core Laboratory of Human Diseases, Chang Gung Memorial Hospital, Keelung, Taiwan; ^4^ Department of Otorhinolaryngology-Head and Neck Surgery, Changhua Christian Hospital, Changhua, Taiwan; ^5^ Department of Medical Research, Chung Shan Medical University Hospital, Taichung, Taiwan; ^6^ Institute of Oral Sciences, Chung Shan Medical University, Taichung, Taiwan; ^7^ Department of Dentistry, Chung Shan Medical University Hospital, Taichung, Taiwan

**Keywords:** single nucleotide polymorphism, WWOX, oral cancer

## Abstract

In Taiwan, oral cancer is the fourth leading cancer in males and is associated with exposure to environmental carcinogens. WW domain-containing oxidoreductase (*WWOX*), a tumor suppressor gene, is associated with the development of various cancers. We hypothesized that genetic variants of *WWOX* influence the susceptibility to oral cancer. Five polymorphisms of *WWOX* gene from 761 male patients with oral cancer and 1199 male cancer-free individuals were genotyped. We observed that individuals carrying the polymorphic allele of *WWOX* rs11545028 are more susceptible to oral cancer. Furthermore, patients with advanced-stage oral cancer were associated with a higher frequency of *WWOX* rs11545028 polymorphisms with the variant genotype TT than did patients with the wild-type gene. An additional integrated in silico analysis confirmed that rs11545028 affects *WWOX* expression, which significantly correlates with tumor expression and subsequently with tumor development and aggressiveness. In conclusion, genetic variants of *WWOX* contribute to the occurrence of oral cancer, and the findings regarding these biomarkers provided a prediction model for risk assessment.

## INTRODUCTION

Oral cancer, a common malignant disease affecting the head and neck region, has a poor prognosis. The incidence of oral cancer is high in Asia, particularly in Taiwan, and it is the fourth common cancer in males [1]. Furthermore, oral cancer was ranked the fifth most common malignancy in Taiwan, annually accounting for more than 2600 deaths in both sexes. Oral squamous cell carcinoma (OSCC) is the most common cancer, accounting for approximately 90% of all oral cancers [2]. Characterizing multiple genetic alterations in OSCC is a critical problem in understanding tumor development and its association with environmental factors including tobacco smoking, alcohol consumption, betel-quid chewing, chronic inflammation, and viral infection [3–6].

The WW domain-containing oxidoreductase (*WWOX*) gene, a tumor suppressor gene, is located on chromosome 16q23 and encompasses the common fragile site FRA16D [7]. *WWOX*, which encodes a 414-amino acid protein, possesses 2 N-terminal WW domains and a high homology domain of the short-chain dehydrogenase/reductase family [8–10]. *WWOX* is emerging as a tumor suppressor that is also involved in metabolic and neurological disorders [11], *In vivo* studies have indicated that the *WWOX* gene is alternatively knocked out in mice, causing Leydig cell development failure in the testis and affecting normal prostate function [12]. However, several studies have reported a loss or downregulation of the *WWOX* protein and homozygous deletion within the *WWOX* locus in multiple malignant neoplasms such as lung cancer, pancreatic adenocarcinoma, oral cancer, ovarian cancer, and renal cell carcinoma [13–21].

Growing evidence emphasizes the importance of genetic variations, which induce cancer by affecting the functions of oncogenes and tumor suppressor genes or enzyme metabolism. The expression of certain genes may be affected by single-nucleotide polymorphisms (SNPs), which are the most common types of DNA sequence variation. Moreover, previous studies have reported the effect of *WWOX* gene polymorphisms on human cancer susceptibility, and they have indicated that genotyping-related SNPs may efficiently predict the risk of cancers and other diseases [22–24]. Highly variable intronic and exonic polymorphisms were observed within *WWOX* in tumor cell lines [25]. In addition, studies have identified several SNPs in *WWOX* as potential risk factors for several cancers such as thyroid carcinomas, esophageal adenocarcinoma, pancreatic and ovarian cancer [22, 26–28]. Genome-wide scan analysis studies conducted on the rs1079635 which is in intron 7 of *WWOX* have also reported that this region demonstrated a strong association with prostate cancer susceptibility [29]. Nevertheless, although the effects of *WWOX* on functional analysis and phenotypic studies are adequately documented, the role of *WWOX* genetic polymorphism in the association between environmental carcinogens and OSCC and the clinicopathological characteristics of OSCC remain poorly investigated. In this study, we used a case-control study with 2 independent cohorts and analyzed 5 SNPs in *WWOX* in addition to investigating the associations between the SNPs and environmental factors. We further investigated the association between genetic factors and oral cancer clinicopathological characteristics.

## RESULTS

### Association between *WWOX* single nucleotide polymorphisms and OSCC

Table 2 shows the results of the statistical analysis of demographic characteristics. Significant differences were observed in the distribution of betel-quid chewing (*p* < 0.001), cigarette smoking (*p* < 0.001), and alcohol consumption (*p* < 0.001) between the controls and patients with OSCC. Table 3 shows genotype distributions and associations between oral cancer and *WWOX* gene polymorphisms. Alleles with the highest distribution frequency for rs11545028, rs12918952, rs3764340, rs73569323, and rs383362 polymorphisms of *WWOX* in both the controls and patients with OSCC were heterozygous for C/C, heterozygous for G/G, homozygous for C/C, homozygous for C/C, and homozygous for G/G, respectively. In these controls, the genotypic frequency of *WWOX* SNP rs11545028, rs12918952, rs3764340, rs73569323, and rs383362 were in the Hardy-Weinberg equilibrium (*p*=0.065, χ2 value: 3.402; *p*=0.093, χ2 value: 2.824; *p*=0.383, χ2 value: 0.759; *p*=0.489, χ2 value: 0.479 and *p*=0.066, χ2 value: 4.947, respectively). Furthermore, after adjustments for several variables, we observed no significant differences in the incidence rates of oral cancer in patients with rs12918952, rs3764340, rs73569323, and rs383362 polymorphisms of *WWOX* compared with those with the wild-type (WT) gene. However, oral cancer patients with the *WWOX* polymorphic rs11545028 T/T and combination of CT and TT genotypes exhibited a 1.824-fold (95% CI: 1.224-2.716) and 1.227-fold (95% CI: 1.022-1.473; both *p* < 0.05) higher risk of OSCC than did patients with the corresponding WT homozygous gene. To clarify the influence of the polymorphic *WWOX* genotypes on the clinicopathological status, such as TNM clinical staging, tumor size, lymph node involvement, and cell differentiation, the distribution frequency of clinical statuses and *WWOX* genotype frequencies in patients with oral cancer were estimated. Regarding the genotypic frequency of the SNPs, *WWOX* rs11545028 demonstrated significant associations with clinical pathological variables in patients with OSCC. The results form Table 4 shown that *WWOX* rs11545028 gene polymorphism is associated with clinical stage (*p*= 0.030) and lymph node metastasis (*p* = 0.010), but no difference was observed in tumor size and cell differentiation (Table 4).

**Table 1:** Variants, position, function, amino acid and changes of observed WWOX sequence variations

	Exon (chromosome position ||)			
	1 (78,099,774)	5 (78,386,878)	7 (78,432,540)	8 (79,211,868)	8 (79,211,923)
nucleotide change	C>T	G>A	C>G	C>T	G>C
mRNA position †	362	901	1,210	1,683	1,738
Protein position ‡		179	282	-	-
domain	-	SDR domain	SDR domain		-
dbSNP (rs number)	rsl 1545028	rsl 2918952	rs3 764340	rs73569323	rs383362
Function	5′UTR	Nonsynonymous	Nonsynonymous	3′UTR	3′UTR
dbSNP allele	-	GCA>ACA	CCA>GCA	-	-
Protein residue	-	Ala>Thr	Pro>Ala	-	-
Codon position	-	1	1	-	-
Heterozygous (%) †	26.8	9.8	17.1	-	40.8

|| CRCh38.p2

† NM_016373.3

‡ NP_057457.1

† HapMap-CHB

SDR domain: short-chain dehydrogenase/reductase domain

**Table 2:** The distributions of demographical characteristics in 1199 male controls and 761 male patients with oral cancer

Variable	Controls (N=1199)	Patients (N=761)	*p* value
Age (yrs)	Mean ± S.D.	Mean ± S.D.	
	53.90 ± 10.02	54.63 ± 11.13	p=0.133
Betel quid chewing			
No	1000 (83.4%)	149(19.6%)	
Yes	199(16.6%)	612 (80.4%)	p < 0.001*
Cigarette smoking			
No	563 (47.0%)	89(11.7%)	
Yes	636 (53.0%)	672 (88.3%)	p < 0.001*
Alcohol drinking			
No	962 (80.2%)	327 (43.0%)	
Yes	237 (19.8%)	434 (57.0%)	p < 0.001*
Stage			
I+II		362 (47.6%)	
III+IV		399 (52.4%)	
Tumor T status			
T1+T2		425 (55.8%)	
T3+T4		336 (44.2%)	
Lymph node status			
NO		509 (66.9%)	
N1+N2+N3		252 (33.1%)	
Metastasis			
MO		752 (98.8%)	
Ml		9(1.2%)	
Cell differentiation			
Well differentiated		117(15.4%)	
Moderately or poorly differentiated		644 (84.6%)	

Mann-Whitney U test or Fisher’s exact test was used between healthy controls and patients with oral cancer.

* *p* value < 0.05 as statistically significant.

**Table 3:** Odds ratio (OR) and 95% confidence interval (CI) of oral cancer associated with *WWOX* genotypic frequencies

Variable	Controls (N=1199) n (%)	Patients (N=761) n (%)	OR (95% Cl)	*p* value
**rsll545028**				
CC	700 (58.4%)	406 (53.4%)	1.00	
CT	447 (37.3%)	300 (39.4%)	1.157 (0.956-1.400)	p=0.134
TT	52 (4.3%)	55 (7.2%)	1.824(1.224-2.716)*	p=0.003*
CT+TT	499 (41.6%)	355 (46.6%)	1.227(1.022-1.473)*	p=0.029*
**rsl2918952**				
GG	1088 (90.7%)	674 (88.6%)	1.00	
GA	111 (9.3%)	82(10.8%)	1.193 (0.882-1.612)	p=0.252
AA	0 (0%)	5 (0.6%)	---	----
GA+AA	111 (9.3%)	87(11.4%)	1.265 (0.940-1.702)	p=0.119
**rs3764340**				
CC	1016 (84.7%)	630 (82.8%)	1.00	
CG	173 (14.4%)	123 (16.2%)	1.147 (0.892-1.475)	p=0.287
GG	10 (0.9%)	8 (1.0%)	1.290 (0.507-3.286)	p=0.593
CG+GG	183 (15.3%)	131 (17.2%)	1.154 (0.903-1.475)	p=0.251
**rs73569323**				
CC	1152 (96.1%)	740 (97.2%)	1.00	
CT	47 (3.9%)	(2.7%)	0.662 (0.389-1.127)	p=0.129
TT	0 (0%)	1 (0.1%)	----	----
CT+TT	47 (3.9%)	21 (2.8%)	0.696 (0.412-1.173)	p=0.171
**rs383362**				
GG	887 (74.0%)	558 (73.3%)	1.00	p=0.596
GT	299 (24.9%)	199 (26.2%)	1.058 (0.859-1.303)	p=0.213
TT	13 (1.1%)	4 (0.5%)	0.489 (0.159-1.508)	p=0.749
GT+TT	312 (26.0%)	203 (26.7%)	1.034 (0.842-1.271)	

The odds ratio (OR) with their 95% confidence intervals were estimated by logistic regression models.

* *p* value < 0.05 as statistically significant.

**Table 4:** Odds ratio (OR) and 95% confidence intervals (CI) of clinical statuses associated with genotypic frequencies of *WWOX* rs11545028 in male oral cancer patients (n=761)

Variable			OR (95% CI)	*p* value
**Clinical Stage**				
**rsll545028**	Stage I+II	Stage III+IV		
	(n=362) (%)	(n=399) (%)		
CC	196 (54.1%)	210(52.6%)	1.00	
CT	148 (40.9%)	152 (38.1%)	0.947 (0.701-1.280)	p=0.781
TT	18(5.0%)	37 (9.3%)	1.919(1.057-3.482)	p=0.030*
**Tumor size**				
**rsll545028**	≦ T2	>T2		
	(n=425) (%)	(n=336) (%)		
CC	226 (53.2%)	180 (53.6%)	1.00	
CT	171 (40.2%)	129 (38.4%)	0.947 (0.701-1.280)	p=0.724
TT	28 (6.6%)	27 (8.0%)	1.211 (0.689-2.128)	p=0.506
**Lymph node metastasis**				
**rsll545028**	No	Yes		
	(n=509) (%)	(n=252) (%)		
CC	278 (54.6%)	128 (50.8%)	1.00	
CT	203 (39.9%)	97 (38.5%)	1.038 (0.754-1.429)	p=0.820
TT	28 (5.5%)	27 (10.7%)	2.0974(1.186-3.698)	p=0.010*
**Cell differentiated grade**				
**rsll545028**	≦ Grade I	>Grade I		
	(n=117) (%)	(n=644) (%)		
CC	59 (50.4%)	347 (53.9%)	1.00	
CT	48 (41.0%)	252 (39.1%)	0.893 (0.590-1.351)	p=0.591
TT	10 (8.5%)	45 (7.0%)	0.765 (0.366-1.602)	p=0.476

Cell differentiate grade:

grade I: well differentiated; grade II: moderately differentiated; grade III: poorly differentiated.

* *p* value < 0.05 as statistically significant.

### Functional analysis of the *WWOX* rs11545028 locus

**Figure 1:**
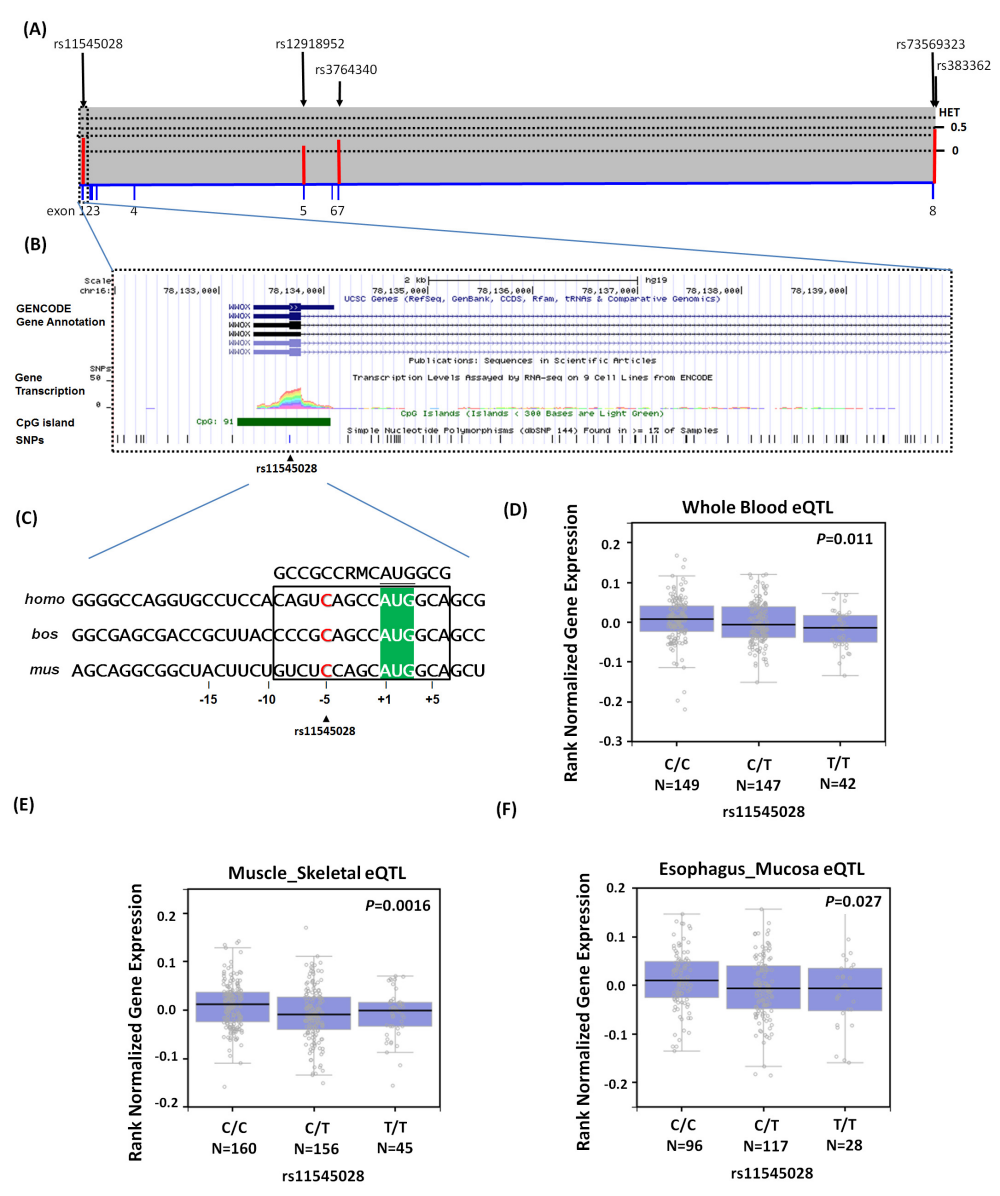
Exon and intron structure of WWOX gene in human and the features of SNPs of WWOX gene (NM_016373.3), which were used to analyze in this study (**A**) Exons are shown by the filled blue boxes and are number 1 to 8 from the chromosome positions chr16:78,133,310 to 79,246,567 (reference genome GRCh37.p13). The lower panel shows population-specific heterozygosity frequencies of this polymorphism in East Asian population (HAPMAP-CHB); and the SNPs of WWOX gene are indicated by the black arrows and labeled reference SNP ID number. (**B**) Expanded view of the ENCODE data for the 5′UTR block containing the WWOX rs11545028 using UCSC genome browser on GRCh37/hg19 assembly. Chromatin State Segmentation track displays chromatin state segmentations by integrating ChIP-seq data using a Hidden Markov Model for H1 embryonic stem cells (color orange), HepG2 hepatocellular carcinoma cells (color green), HUVEC umbilical vein endothelial cells (color deep blue), HSMM (color blue), skeletal muscle myoblasts (color yellow), NHEK epidermal keratinocytes (color purple), and NHLF lung fibroblasts (color red). CpG islands are typically common near transcription start sites and may be associated with promoter regions. (**C**) Upstream open reading frames in *WWOX* transcripts of human (*homo, NM_016373.3*), cow (*bos, NM_001078092*), and mouse (*mus, NM_019573.3*) sequences shown in this alignment. Initiation codons of *WWOX* protein are highlighted by green background color. Consensus residues of the core Kozak context (residues at -9 or +3) are above this alignment in box, where M denotes A or C; R denotes A or G. (**D**-**F**) Expression quantitative trait locus association between rs11545028 and WWOX expression in (D) whole blood, (E) muscle skeletal and (F) esophagus mucosa (GTEx data set). Numbers in parentheses indicate the number of cases.

We also investigated whether rs11545028 was associated with the differential expression of *WWOX* as a preliminary assessment of the putative functional role of the SNP. We obtained human *WWOX* from the NCBI gene database, selected its respective longest transcript, and defined its promoters as 1-kb upstream to 1-kb downstream of the predicted transcription start sites (Figure 1A). Moreover, we identified the putative functional role of rs11545028, as indicated by the functional annotations in the ENCODE data. We determined that rs11545028 was situated at a locus with TF binding, histone modification patterns, DNase hypersensitivity, and CpG islands that were characterized as promoters or enhancers in several cell types (Figure 1B). The effect of rs11545028 may be attributed to the suboptimal Kozak context surrounding the initiation codon of upstream open reading frames of human *WWOX* (Figure 1C), which enables the modulation of initiation rates in response to the translational status. In addition, the GTEx database revealed a statistically significant downregulation of *WWOX* mRNA expression in the whole blood, muscle skeletal and esophagus mucosa of rs11545028-variant genotypes (CT or TT) compared with that of the WT homozygous CC genotype (*p* = 0.011, *p* = 0.0016 and *p* = 0.027, respectively) (Figure 1D-1E).

### Functional analysis of the *WWOX* rs11545028 locus in clinical sample

**Figure 2:**
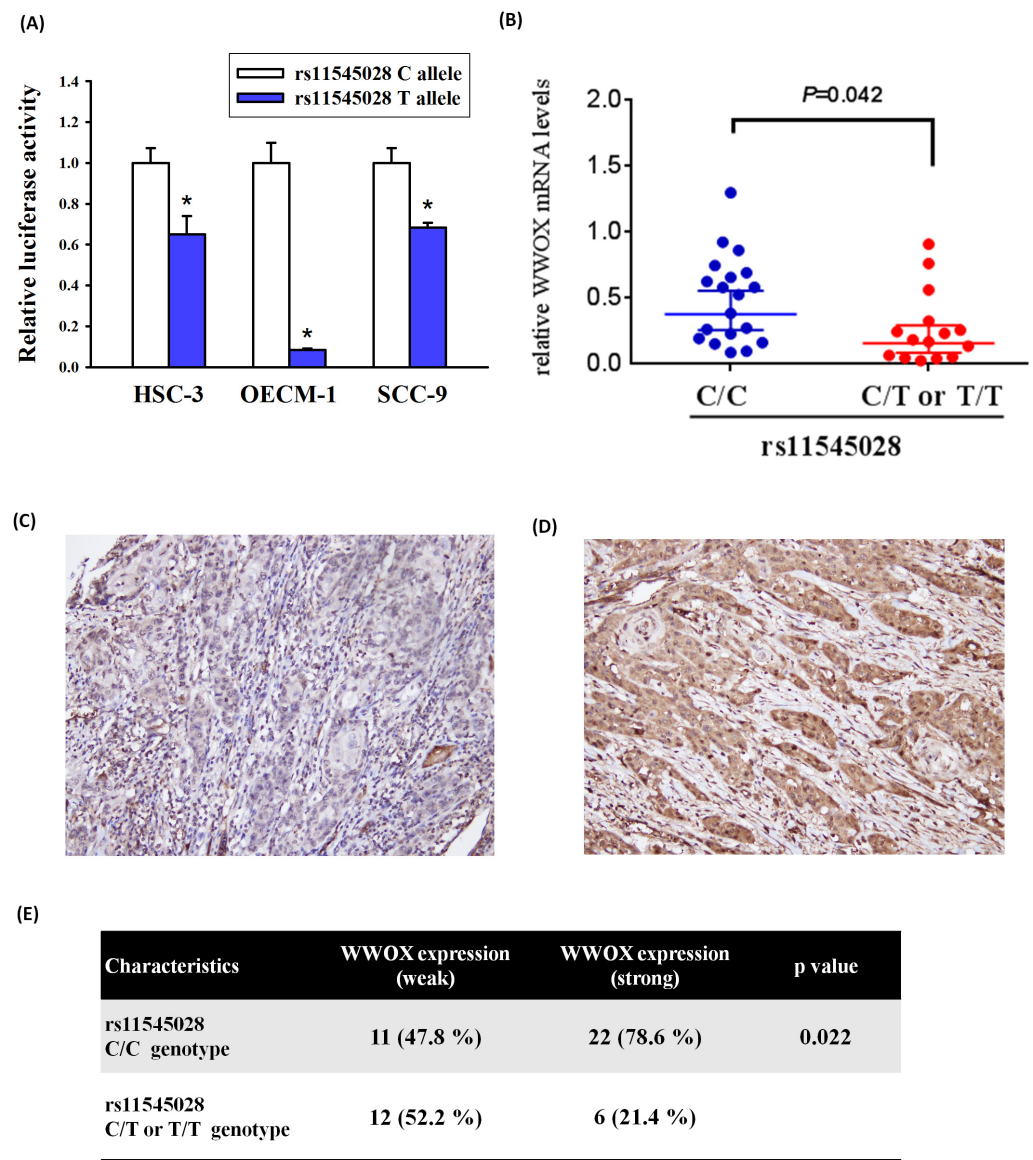
WWOX expression was correlated with rs11545028 genotypes in oral cancer patients (**A**) Two luciferase reporter vectors with either the rs11545028 C allele or the rs11545028 T allele were transfected to HSC-3, OECM-1 and SCC-9 oral cancer cells lines. Data are mean values with standard deviation from at least three independent experiments. (**B**) WWOX mRNA expression in cancer tissue of 34 OSCC patients was analyzed by quantitative real time-PCR assay. Numbers in parentheses indicate the number of cases. (**C**) Weak cytoplasmic WWOX expression in OSCC. (**D**) Strong cytoplasmic WWOX expression in OSCC. (**E**) The OSCC specimens with rs11545028 C/C have higher WWOX expression, while specimens with rs11545028 C/T or T/T have lower WWOX expression (p=0.022).

To determine the functional effect of the rs11545028 polymorphism on *WWOX* expression, we generated luciferase reporter vectors with either the rs11545028 C allele or the rs11545028 T allele. We used these vectors for transfection of HSC-3, OECM-1 and SCC-9 oral cancer cells lines. As shown in Figure 2A, the vectors with the rs11545028 T allele had significantly lower luciferase activity compared to the vectors with the rs11545028 C allele among these three cell lines (p<0.05). Furthermore, to realize correlation between the mRNA, protein level of WWOX and rs11545028 polymorphism, quantitative real time-PCR (qPCR) and Immunohistochemical (IHC) staining were used to analyze WWOX mRNA and protein expression in cancer tissue of 34 and 51 OSCC patients, respectively. We found that OSCC patient who carry C/T or T/T of rs11545028 polymorphism have significantly lower mRNA levels of WWOX compare to C/C genotype (Figure 2B). Furthermore, when WWOX expression was classified into a two-tier grading system of weak (−/1+) (Figure 2C) and strong (2+/3+) (Figure 2D) WWOX staining, our analysis shown that specimens with rs11545028 C/C have higher WWOX expression, while specimens with rs11545028 C/T or T/T have lower WWOX expression (p=0.022) (Figure 2E). Overall, rs11545028 C to T substitution might affect the translational initiation and reduce WWOX mRNA and protein expression and the risk of oral cancer.

## DISCUSSION

Several studies have suggested that chromosome 16q23 contains a tumor suppressor gene involved in multiple tumor types. *WWOX* was mapped to this region, and the loss of function of *WWOX* in cancer cells was associated with mucinous histologies and a poor prognosis, suggesting that *WWOX* suppresses tumor progression [30]. Recent studies have reported that *WWOX* polymorphism is associated with the susceptibility to several carcinomas including lung, breast, bladder, colorectal, and pancreatic cancers [31, 32]. Five SNPs were included in a case-control study with 2 independent cohorts design. One of the SNPs (rs11545028) is located in exon 1 of *WWOX*. Our data reveal an increased risk of OSCC among patients with the *WWOX* polymorphic rs11545028 T/T compared with those with homozygous C/C. Only few studies have examined the functional role of rs11545028, and its functional importance has yet to be examined. An association of the risk of OSCC with the location of the analyzed variant is proposed.

In lung cancer, exonic polymorphisms within *WWOX* were revealed to exhibit a high incidence of the deletion of exon 6-8, which may result from amino acid changes and thus the loss of the tumor suppression function of *WWOX*. Furthermore, missense polymorphisms of *WWOX*, including Arg−314→His, Lys−182→Glu, Arg−120→Trp, and Thr−111→Ser, were detected in blood specimens from 15 and 34 patients with ovarian and colorectal cancers, respectively, but not in healthy participants [25]. By contrast, in our study, 2 of the missense SNPs located in exon 6-8 did not confer a risk of OSCC. In contrast to other tumors, the effect of missense polymorphisms in exon 6-8 did not alter the frequency of DNA strand breakage in OSCC, and this factor might be associated with the cancer type. Therefore, we suggested the presence of another major regulating mechanism associated with the downregulation of *WWOX* expression in OSCC.

Notably, we observed that the nonsense polymorphism rs11545028 C > T located in exon 1 conferred an increased risk of OSCC. Previous studies have reported that rs11545028 (C121T) in the data set indicated no significant difference between each tumor cell line and normal cell lines, even when the frequency of T/T in patients was lower. In this study, we determined whether the genetic variant rs11545028 C > T contributed to oral cancer susceptibility. Functional annotations from the ENCODE data indicate that rs11545028 is located in the region of an open chromatin, which probably corresponds to the promoters and CpG islands of *WWOX*. Several studies have documented the importance of transcriptional regulation between *WWOX* polymorphisms and cancer risk [23, 33]. Moreover, the common modifications of epigenetic changes in chromatin include DNA methylation, which has been considered a crucial mechanism underlying the inactivation of tumor suppressor genes as well as the loss of heterozygosity and mutation. An abnormal DNA methylation mainly occurs in the promoter region (CpG islands), which is associated with the transcriptional inactivation of tumor suppressor genes during tumor progression. The methylation rate of the *WWOX* promoter has been reported to be associated with the loss of WWOX expression in breast, lung, bladder, pancreatic, and prostate cancers [14, 32, 34]. The CpG methylation status in the *WWOX* promoter region was significantly higher in late-stage epithelial ovarian cancer tissues than in early stage epithelial ovarian cancer tissues [35]. In head and neck squamous cell carcinoma, it has also been reported that WWOX expression was decreased by miR-134 and promoter methylation [36, 37]. Liu et al. showed miR-134 expression contributes to head and neck carcinogenesis by targeting the WWOX [36]. Moreover, Ekizoglu et al. reported that decreased WWOX expression in advanced-stage tumor samples or in tumors with OSCC was associated with methylation of the WWOX promoter region [37]. Pimenta et al. also shown that the WWOX gene alteration is an early genetic alteration and may contribute to oral carcinogenesis [38]. Nevertheless, the frequency of the methylation of the *WWOX* promoter was not explained in our study, and the methylation rate must be examined further. Critical evidence indicates the importance of the methylation of the *WWOX* promoter. The highest methylation at the CpG site (approximately 60%) was observed in the promoter region (−328 to −41 bp) and exon 1 (−27 to +334 bp) of *WWOX* [39]. Furthermore, an aberrant methylation of these *WWOX* regions may occur at the early stage of cancer and, more precisely, at the advanced stage of esophageal squamous cell carcinoma, thus coinciding with the rs11545028 (C121T) region. This observation suggests that *WWOX* methylation is a critical event in the development of OSCC and that silencing through *WWOX* methylation is a pivotal mechanism underlying *WWOX* inactivation.

In a previous study, rs11545028 was predicted to lie within the Kozak translation initiation site, which comprises 6−8 nucleotides surrounding the initiation codon [40]. Studies conducted on the optimal Kozak sequence at positions −3 and +4 have proposed a valuable method for determining gene expression. However, the Kozak sequence has been increasingly demonstrated to be capable of altering translational machinery in response to the regulation of gene expression [41, 42]. A recent study revealed that an SNP located at position −1C/T in the Kozak sequence of *CD40* was highly meaningful because the *CD40*expression levels were significantly higher in −1C/C carriers than in −1C/Tand −1T/T carriers [43]. Consistent with this observation, the less consensus Kozak sequence involving the nucleotide T at position −4 may markedly affect protein expression [44]. Figure 1C shows that the *WWOX* polymorphism rs11545028 at position −5 that involves the original consensus Kozak sequence contains the nucleotide C. Previous studies have demonstrated an association between the C allele of the Kozak polymorphism and gene expression both *in vitro* and *in vivo*. In the current study, the sequence containing the nucleotide C at position −5 more closely approximated the Kozak consensus, suggesting that the mRNA with the nucleotide T at 121 was associated with a markedly diminished efficiency. The GTEx database also revealed a significant drop in the *WWOX* mRNA expression in carriers of a genotype involving the variant T at rs11545028. In addition, we observed a high frequency of the homozygous 121 TT genotype and its combination with the heterozygous *WWOX* CT in patients, suggesting that changes in the translation initiation rate generally explains the differences in protein expression among the participants. The *WWOX* expression also suppresses tumor growth and induces cell apoptosis [45]. We observed that rs11545028 was associated with a higher risk of stage III and IV cancers, lymph node metastasis, and the cell differentiation grade. Overall, our findings suggest that the rs11545028 T allele reduced the translation initiation rate, which subsequently reduced the *WWOX* expression, thus contributing to a more aggressive phenotype in OSCC.

In conclusion, examining the complete medical information and conducting additional bioinformative analyses of a high number of patients provided comprehensive evidence of *WWOX* polymorphism in OSCC. Our results suggest that the *WWOX* polymorphic rs11545028 C/T in the suboptimal Kozak context is associated with clinical statuses and susceptibility to OSCC. The coeffects of *WWOX* polymorphism and environmental carcinogens markedly facilitate OSCC development. Overall, our analyses provide deeper insights into naturally occurring TIS variants. Comprehensive data on such types of variant are required for developing therapeutic approaches that can eventually ameliorate the clinical phenotype in patients harboring the corresponding lesions.

## MATERIALS AND METHODS

### Patient specimens

In 2007–2014, for the case group, we recruited 761 male patients at Chung Shan Medical University Hospital in Taichung and Changhua Christian Hospital in Changhua, Taiwan. For the control group, we randomly chose 1199 male non-cancer individuals from Taiwan Biobank and these control groups had neither self-reported history of cancer of any sites. For both groups, we administered a questionnaire to obtain information on their exposure to betel quid chewing, tobacco use, and alcohol consumption. Medical information of the patients, including TNM clinical staging, primary tumor size, lymph node involvement, and histologic grade, was obtained from their medical records. All participants provided written consent, and the Chung-Shan Medical University Hospital ethics committees approved the research protocol and informed consent was obtained from all subjects (CSMUH No: CS13214-1). All the methods applied in the study were carried out in accordance with the approved guidelines.

### DNA extraction

DNA was extracted from buffy coats (white blood cells) using a QIAamp DNA blood mini kits (Qiagen, Valencia, California) as described in detail previously [46]. DNA was dissolved in TE buffer and used as the template in polymerase chain reactions

### SNP selection and genotyping

In this study, the selection of 5 well-characterized common polymorphisms from *WWOX* gene is based on their wide associations with the development of cancer (Table 1, Figure 1A) [23–26, 33]. We included rs11545028 in the 5′UTR region. Rs12918952 and rs3764340, which are located in the exon of WWOX, were selected in this study since these 2 SNPs may result from amino acid changes and thus the loss of the tumor suppression function of WWOX [25]. The allelic discrimination of *WWOX* rs11545028, rs12918952, rs3764340, rs73569323, and rs383362 polymorphisms were assessed using an ABI StepOne TM Real-Time PCR System (Applied Biosystems, Foster City, CA) and analyzed using SDS v3.0 software (Applied Biosystems, Foster City, CA) as previously described [47].

### Construction of luciferase reporter plasmids

A luciferase reporter plasmid encompassing the major allele (C) and minor allele (T) of rs11545028 in the promoter region of the WWOX gene was cloned into the pGL3-Enhancer Luciferase Reporter Vectors (Promega Corp., Madison, WI, USA), according to manufacturer instructions. The vectors were sequenced to confirm the orientation and integrity.

### Transient transfections and luciferase assay

HSC-3 cells were purchased by the Japanese Collection of Research Bioresources Cell Bank (JCRB, Shinjuku, Japan) [48]. SCC-9 cells were purchased from and validated by the American Type Culture Collection (ATCC, Manassas, VA, USA). Both cell lines maintained in DMEM/F12 supplemented with 10% FBS, 400 ng/ml hydrocortisone and 0.1 mM non-essential amino acids (NEAA; Life Technologies). OECM-1 cells were obtained from Dr Meng's group where the cell line is originally established and authenticated and maintained in RPMI (Gibco) supplemented with 10% FBS [49]. All the cells were cultured and maintained at 37 °C in a 5% CO_2_ and 95% air atmosphere. Each cell was seeded per well in a 24-well plate, and each well was transfected with 0.75 μg of the vector DNA containing either the rs11545028 C allele or the rs11545028 T allele by using the Lipofectamine 2000 reagent (Invitrogen, Carlsbad, CA, USA), according to manufacturer instructions. Cells were collected 48 h after transfection and analyzed for luciferase activity by using the Luciferase Reporter Assay System (Promega, Madison, WI, USA). All transfections were performed in duplicate and repeated three times.

### RNA preparation, TaqMan quantitative real-time PCR

Total RNA was isolated from oral cancer tissues using RNeasy Mini Kit (Qiagen, Valencia, CA, USA). Quantitative real-time PCR analysis was performed using TaqMan one-step PCR Master Mix (Applied Biosystems, Foster City, CA, USA). Total cDNA (2 μg) was added per 9 μl reactions with WWOX or GAPDH primers and TaqMan probes. The WWOX (Hs03044790_m1) and GAPDH (Hs99999905_m1) primers and probes were designed using commercial software (ABI PRISM Sequence Detection System; Applied Biosystems, Foster City, CA, USA) as previously described [50].

### Immunohistochemistry

OSCC tissue microarray block slides were deparaffinised, as stated in our previous study [51]. The slides were incubated with 1:200 diluted anti-WWOX antibodies (Santa Cruz Biotechnology, Santa Cruz, CA, USA) for 60 min at room temperature. After thoroughly washing with PBS, the conventional streptavidin–biotin peroxidase method (LSAB Kit K675; Dako, Copenhagen, Denmark) using 3,3′-diaminobenzidine (DAB) was employed for assessing signal development. Two pathologists blinded to the clinical outcomes semiquantitatively assessed WWOX expression based on the staining intensity; they independently scored sections through light microscopy.

### Statistical analysis

Mann–Whitney U-test and Fisher's exact test were used to compare the age differences and demographic characteristic distributions between the controls and patients with oral cancer. The odds ratio and 95% CIs of the association between the genotype frequencies and oral cancer risk and the clinical pathological characteristics were estimated using multiple logistic regression models. *p* < 0.05 was considered significant. The data were analyzed on SAS statistical software (Version 9.1, 2005; SAS Institute, Cary, NC).

### Bioinformatics analysis

We used several semiautomated bioinformatics tools for assessing whether rs11545028 or its related genetic variants were associated with a putative function that might affect patient outcomes. HaploReg [52] v4 and Genotype-Tissue Expression (GTEx) [53] from the Encyclopedia of DNA Elements (ENCODE) [54] project were used for identifying the regulatory potential of candidate functional variants to examine factors of interest such as transcription factor (TF)–chromatin immunoprecipitation signals, DNase peaks, DNase footprints, and predicted DNA sequence motifs for TFs. The GTEx data were used for identifying the associations between the SNPs and whole blood-specific gene expression levels. Moreover, the publicly available cBioPortal for Cancer Genomics [55] and UCSC Cancer Genomics Browser [56] for hepatocellular adenocarcinomas were used for analyzing *WWOX* expression, DNA methylation, molecular features, and clinical outcomes.
